# MicroRNA 3113-5p is a novel marker for early cardiac ischemia/reperfusion injury

**DOI:** 10.1186/s13000-019-0894-1

**Published:** 2019-10-31

**Authors:** Yuanyuan Chen, Xing Ye, Fengping Yan

**Affiliations:** 1 Department of Forensic Medicine, School of Basic Medical Sciences, Gannan Medical College, 1 Yixueyuan Road, Zhanggong District, Ganzhou, Jiangxi 341000 People’s Republic of China; 2 Academy of Forensic Science, Ministry of Justice of China, Shanghai, 200063 People’s Republic of China; 3 Department of Forensic Medicine, School of Basic Medical Sciences, Fudan University, Shanghai, 200032 People’s Republic of China; 4 Key Laboratory of Prevention and Treatment of Cardiovascular and Cerebrovascular Diseases of Ministry of Education, Gannan Medical College, Ganzhou, 341000 Jiangxi China

**Keywords:** Ischemia/reperfusion injury, Heart, Cardiac biomarkers, microRNA 3113-5p

## Abstract

**Background:**

Ischemia/reperfusion (I/R) injury of heart is one of the major causes of acute cardiac injury, which may result in worsening or even loss of heart function. With novel microRNAs being evolutionarily discovered, numbers of them remained functionally unknown. We aimed to discover novel microRNAs with therapeutic or diagnostic potential in the setting of early cardiac I/R injury.

**Methods:**

Cardiac electrical activity, biochemical detection and histopathology analysis were performed to reveal early changes of cardiac I/R injury. A microRNA array was performed to screen differential microRNAs in the mouse model of cardiac I/R injury. The differentially expressed microRNAs were validated in cardiac tissues and in serum samples.

**Results:**

The abnormality in electrocardiogram and increases in serum cTnI levels suggested the successful establishment of cardiac I/R injury in mice. A total of 1882 microRNAs were identified, of which 11 were significantly down-regulated and 41 were significantly up-regulated at 3 h post reperfusion. microRNA 223-3p and microRNA 3113-5p were among the mostly altered microRNAs and were validated to be up-regulated within the early hours of I/R injury in heart tissues. In the circulating system, cTnI, a sensitive marker of cardiac injury, or microRNA 223-3p only increased within the first 6 h post I/R injury. However, microRNA 3113-5p stably increased in the serum, keeping an increase of 2.5-fold throughout the 24 h. In the human serum samples, microRNA 3113-5p was detected to be significantly upregulated as soon as 3 h after I/R stimuli and kept significantly higher levels within the 48 h.

**Conclusion:**

This is the first study that reported the functional roles of microRNA 3113-5p in cardiovascular system. Our data suggested that cardiac microRNA 3113-5p might be a useful target for therapeutic purposes and circulating microRNA 3113-5p might serve as a stable marker for early diagnosis of cardiac I/R injury.

## Background

Ischemia/reperfusion injury (I/R injury) is defined as initial lack of oxygen due to deprivation of blood flow and the following restoration of blood supply from the occluded vessel. I/R injury is a critical mechanism of organ injury [1], and a central cause of tissue injury during various medical conditions, including surgical procedures, organ transplantation, cardiovascular diseases (i.e. myocardial infarction, and circulatory shock) and toxic insults [2]. During the development of I/R injury, a surge of reactive oxygen and nitrogen species as well as inflammatory cascades bursts and eventually triggers organ damage [3]. Other processes such as protein post-translational modifications, lipid oxidations, and DNA breakage are also common mechanisms that aggravate a chain of deleterious responses which eventually result in dysfunction of endothelial cells, neutrophils transmigration to the insulted endothelium, burst of inflammatory cytokines, calcium iron overload, and eventual cell death [4]. Long-term insult by I/R injury could lead to irreversible injury to heart and many studies have demonstrated various therapeutic strategies against long-term I/R injury [5]; however, early diagnosis and treatment of I/R injury, which receives scanty attention, is advantageous over late-stage intervention and thus mandates more research focus.

Recently, microRNAs have been shown to implicate in the pathophysiology of cardiac I/R injury [1]. Multiple microRNAs have been reported to have diagnostic values or therapeutic potentials [6]. For example, microRNA 21 contributed to cardiac diseases by triggering mitogen-activated protein kinase (MAPK) activity in fibroblasts [7]. MicroRNA 21 lowers blood pressure in spontaneous hypertensive rats by upregulating mitochondrial translation [8]. microRNA 223-3p is another widely documented microRNA which has been reported to regulate cardiac fibrosis after myocardial infarction by targeting RAS p21 protein activator 1 (RASA1) [9], and regulated expression of voltage-gated K+ channel Kv4.2 in acute myocardial infarction [10].

Due to the nature of external secretion and functional importance in heart diseases, multiple microRNAs have been anticipated as non-invasive biomarkers. So far, more than 200 microRNAs have been considered as heart-specific with many of them being identified to release into the circulatory system during ischemic injury. It is reported that the combination of microRNA 199a-3p, microRNA 208a-3p, microRNA 122-5p, and microRNA 223-3p has a good diagnostic performance for hypertension [11]. Elevated plasma microRNA 223 content associated with the severity of coronary heart disease [12]. Circulating microRNA 1, 499-5p, and microRNA 133a or the cardiomyocyte-specific microRNA 208b have been reported to instantly increase in patients with the onset of ST-elevated myocardial infarction (STEMI) and their levels peak within 12 h after disease onset [13]. However, despite the numerous reports documenting the potential of microRNAs serving as biomarkers for I/R injury, only limited number of microRNAs have been applied in clinical trials, implicating there is still space to identify novel microRNAs.

One fact is that microRNAs keep expanding and undergo evolutionarily identification [14]. With techniques deeply sequencing microRNAs, some new microRNAs with functionality unknown have been identified [15, 16]. By using the newly refreshed database, an updated microRNA array could provide a panel of microRNAs, some of which might represent new ones that may otherwise remain functionally unknown [15, 16]. The present study aimed to perform an updated microRNA array analysis of heart tissues undergoing I/R injury in order to discover novel microRNAs. The identified microRNAs were then validated through quantitative analysis. Our data provided novel biomarkers for diagnosis and treatment of early cardiac I/R injury.

## Methods

### Experimental protocol

Male C57BL/J mice (~ 25 g) were initially anesthetized with 5% chloral hydrate and injected with heparin intravenously at a final concentration of 500 U/kg. Mouse heart was then exposed and perfused according to Langendorff with oxygenated, normothermic Krebs-Henseleit buffer as previously described [17]. For the purpose of this study, different reperfusion time was applied to the indicated group of mice. A time-matched nonischemic control group was aerobically reperfused for 190 min. For the details of inducing I/R injury, the left anterior descending coronary artery (LAD) was undergoing continuous occlusion for 45 min to induce regional ischemia. Thereafter, the occluded LAD was reopened to let perfusion with different hours (1 h, 3 h, 6 h, 12 h, or 24 h). Heart rate and coronary flow were real-time monitored during the perfusion periods in all groups. To confirm successful ligation of the LAD, a Data Analysis System (BL-420; TME Technology, Chengdu, Sichuan, China) was used to record the electrocardiogram (ECG) during the I/R period. Samples from all groups (*n* = 6/group) were immediately frozen in liquid nitrogen for subsequent microRNA isolation. To further assess tissue injury, heart release of cardiac Troponin I (cTnI) was measured using a cTnI ELISA kit (Life Diagnostics Inc., West Chester, PA, USA) from serum samples. In brief, to collect mice serum samples, mice were anesthetized and their hearts were exposed. Following exposure of hearts, the LAD was directly ligated and then reopened for different hours (1 h, 3 h, 6 h, 12 h, or 24 h). In a second series of experiments, the LAD-supplied risk regions of the left ventricle were immediately sampled at the end of 3-h reperfusion and subject to further analysis of differentially expressed microRNAs. For sampling, a single oblique cut was made from the origin of the LAD toward the right side of the apical area. In this way, the samples involve the majority of the left ventricle anterior wall as well as the apex of the heart.

### Histopathology analysis

The left ventricle from the LAD-supplied zone was fixed in 4% paraformaldehyde, dehydrated, and embedded in paraffin. Paraffin-embedded tissues were then cut into 4 μm-thick slices, which were then stained with hematoxylin & eosin (HE) for histological examination. Briefly, after deparaffinization and rehydration, myocardial sections were sequentially stained with eosin for cytoplasm staining and hematoxylin for nucleus staining. Digital images were obtained at × 200 magnification by microscopy (Olympus, Tokyo, Japan).

### Isolation of microRNAs from serum and cardiac tissues

Circulating miRNAs were isolated with the miRNeasy mini kit (Qiagen, Hilden, Germany) according to the manufacturer’s instructions. RNAs from heart tissues were isolated using TRIzol® (Life Technologies, Carlsbad, CA, USA) according to the manufacturer’s instructions. RNA purity and concentration were assessed by spectophotometry at 260 and 280 nm (NanoDrop Products, Wilmington, DE, USA) equipped with a 2100 Bioanalyzer (Agilent Technologies, Santa Clara, CA, USA).

### Microarray analysis of microRNA expression

For the purpose of microarray analysis, all microRNAs from heart samples were initially labeled with the microRNA Complete Labeling and Hyb kit system (Agilent Technologies). Labeled samples were then completely vacuum dried at a medium-high temperature (45 °C) and hybridized onto the surface of a mouse microRNA Microarray (Agilent Technologies) in a microarray hybridization chamber (Agilent Technologies) according to previous regulations [18]. The scanning of each array was in accordance with a previous published protocol [19]. Statistical analysis was performed and explicitly described below. Briefly, the fluorescent signal intensity data represented microRNA expression and changes in gene expression were determined as ratios of signal intensity values. For visual comparison and representation of both down- and upregulation, data were processed and depicted as log2 changes.

Analysis of differentially expressed microRNAs were done using Feature Extraction software (Agilent Technologies) as previously described [20]. All individual microRNAs were represented by 20 different probes on the array. A microRNA was considered to be detectable when at least 1 probe from all the 20 probes was detected. The final gene signal equals to the sum of all signals of each individual probe. Using a two-tailed two-sample unequal variance Student’s t-test, the *P* value was used as a determinate to find significantly expressed microRNAs. A corrected P value was calculated for each microRNA to control the false discovery rate (FDR) using the Benjamini and Hochberg multiple testing correction protocol. MicroRNA expression ratios with *P* values less than 0.05 and log2 changes of less than − 1.0 or log2 changes of more than 1.0 were considered as significant repression or overexpression, respectively.

### Reverse transcription quantitative polymerase chain reaction (RT-qPCR) analysis

Reverse transcription of the total RNA into cDNA was performed using the High Capacity RNA-to-cDNA transcription kit (Applied Biosystems), according to the manufacturer’s instructions. The RT-qPCR assays were performed using the TaqMan MicroRNA Reverse Transcription Kit (Applied Biosystems) following with the TaqMan MicroRNA Assays (Applied Biosystems) according to the manufacturer’s instructions on a 7500 Real-Time RT-qPCR system (Applied Biosystems). The RT-qPCR conditions were as follows: 95 °C for 10 min, and 40 cycles of 95 °C for 13 s and 60 °C for 60 s. The relative expression levels of serum microRNAs were normalized to the relative expression level of U6. The relative expression of microRNAs was calculated using the 2-ΔΔCt equation [21]. Primers used in this study were listed in Table 1. The U6 primer sequences and universal reverse primers were provided by the kits.

**Table 1:** Forward primer sequences used in this study

miRNAs	Primer Sequences 5′-3′
mmu-miR-146a-5p	TGAGAACTGAATTCCATGGGTT
mmu-miR-150-5p	TCTCCCAACCCTTGTACCAGTG
mmu-miR-202-3p	AGAGGTATAGCGCATGGGAAGA
mmu-miR-30b-5p	TGTAAACATCCTACACTCAGCT
mmu-miR-3968	CGAATCCCACTCCAGACACCA
mmu-miR-1224-5p	GTGAGGACTGGGGAGGTGGAG
mmu-miR-188-5p	CATCCCTTGCATGGTGGAG
mmu-miR-1895	CCGAGGAGGACGAGGAGGA
mmu-miR-1892	ATTTGGGGACGGGAGGGAGGAT
mmu-miR-327	ACTTGAGGGGCATGAGGAT
mmu-miR-3113-5p	GTCCTGGCCCTGGTCCGGGTCC
mmu-miR-709	GAGGCAGAGGCAGGAGGAT
mmu-miR-223-3p	TGTCAGTTTGTCAAATACCCCA
mmu-miR-154-5p	TAGGTTATCCGTGTTGCCTTCG
mmu-miR-5121	AGCTTGTGATGAGACATCTCC

We also analyzed the relative expression of predicted target genes of microRNAs. *Gapdh* was used as the internal control gene for normalization. The primers used were listed in Table 2.

**Table 2:** Primer sequences for target genes

Gene	Primers
*Zfp933*	Forward 5′-CAGGCAGGCTTCTCCTTATT-3’
	Reverse 5′-CCTGGTCTACAGAGTGAGTTTC-3’
*Gapdh*	Foward 5′-TGCGACTTCAACAGCAACTC-3’
	Reverse 5′-ATGTAGGCCATGAGGTCCAC-3’

### Statistical analysis

Microarray and RT-qPCR data were presented as means ± standard error of mean (SEM). The Students’ *t*-test was used to compare differences of means between groups. The one-way analysis of variance (ANOVA) was used to evaluate differences among ≥3 groups, which was followed by least-significant-difference (LSD) post hoc test for comparisons within groups. *P* < 0.05 was considered to be statistically significant.

## Results

### Early cardiac I/R injury lacks biochemical biomarkers and presented nonspecific histologic changes

**Fig. 1:**
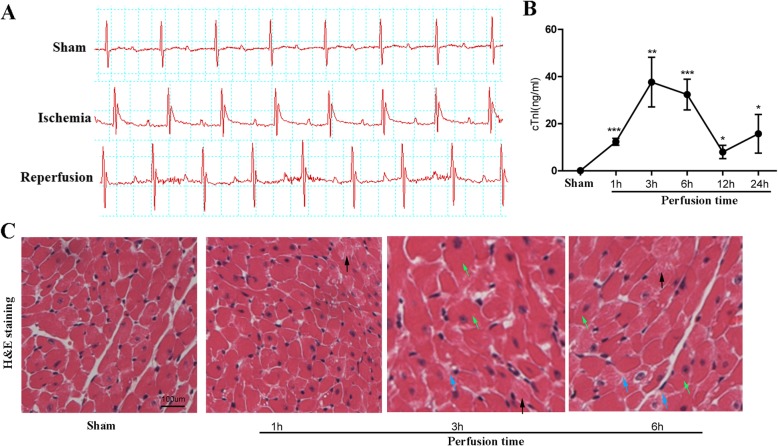
Establishment of a murine model bearing cardiac ischemia/reperfusion (I/R) injury. **a** Electrocardiogram monitoring of mice with (ischemia group) or without (Sham group) coronary artery occlusion. Mice after re-opening of the left anterior descending (LAD) artery (perfusion group) were also subject to ECG monitoring. **b** ELISA detection of serum cTnI levels in Sham group of mice and in mice with distinct perfusion time. **c** Hematoxylin & Eosin (HE) staining of heart tissues were performed. Myocardial cells in the sham group were well-ordered with regular structure, while myocardial cells from the I/R injured mice were swelling in the plasma (black arrow) and showed slight interstitial edema (blue arrow) or occasional cell hypertrophy (green arrow) after 3 or 6 h’ reperfusion. *, *p* < 0.05 vs. Sham

Initially, we occluded the left coronary for 45 min and then perfused the artery for different hours (1 h, 3 h, 6 h, 12 h and 24 h) to establish a murine model of cardiac I/R injury. ECG monitoring showed that the mice with LAD occlusion (ischemic mice) exerted remarkable elevation of ST segment, while the ST segment in the sham group of mice remained at the baseline. After 45 min of occlusion, the LAD was re-opened and ECG monitoring showed that the ST segment declined to baseline after 3 h’ perfusion (Fig. 1a), conforming to the manifestation of I/R injury. Moreover, the serum levels of cTnI, a sensitive cardiac injury marker, dramatically increased in the first 3 h after reperfusion; thereafter it dropped but remained significantly higher in the I/R injured mice than that in Sham mice (Fig. 1b). HE staining is the gold standard for diagnosing most of heart diseases. However, despite the significant elevation of cTnI levels during the initial 3 h’ perfusion, histological analysis of heart tissues showed that myocardial cells in the sham group were well-ordered with regular structure. Myocardial cells from the I/R injured mice were swelling in the plasma (black arrow) and showed slight interstitial edema (blue arrow) or occasional cell hypertrophy (green arrow) after 3- or 6-h’ reperfusion. The non-specific histopathological manifestations suggested that histological analysis did not aid in making a diagnosis of cardiac I/R injury.

### microRNA array identified multiple novel microRNAs

**Fig. 2:**
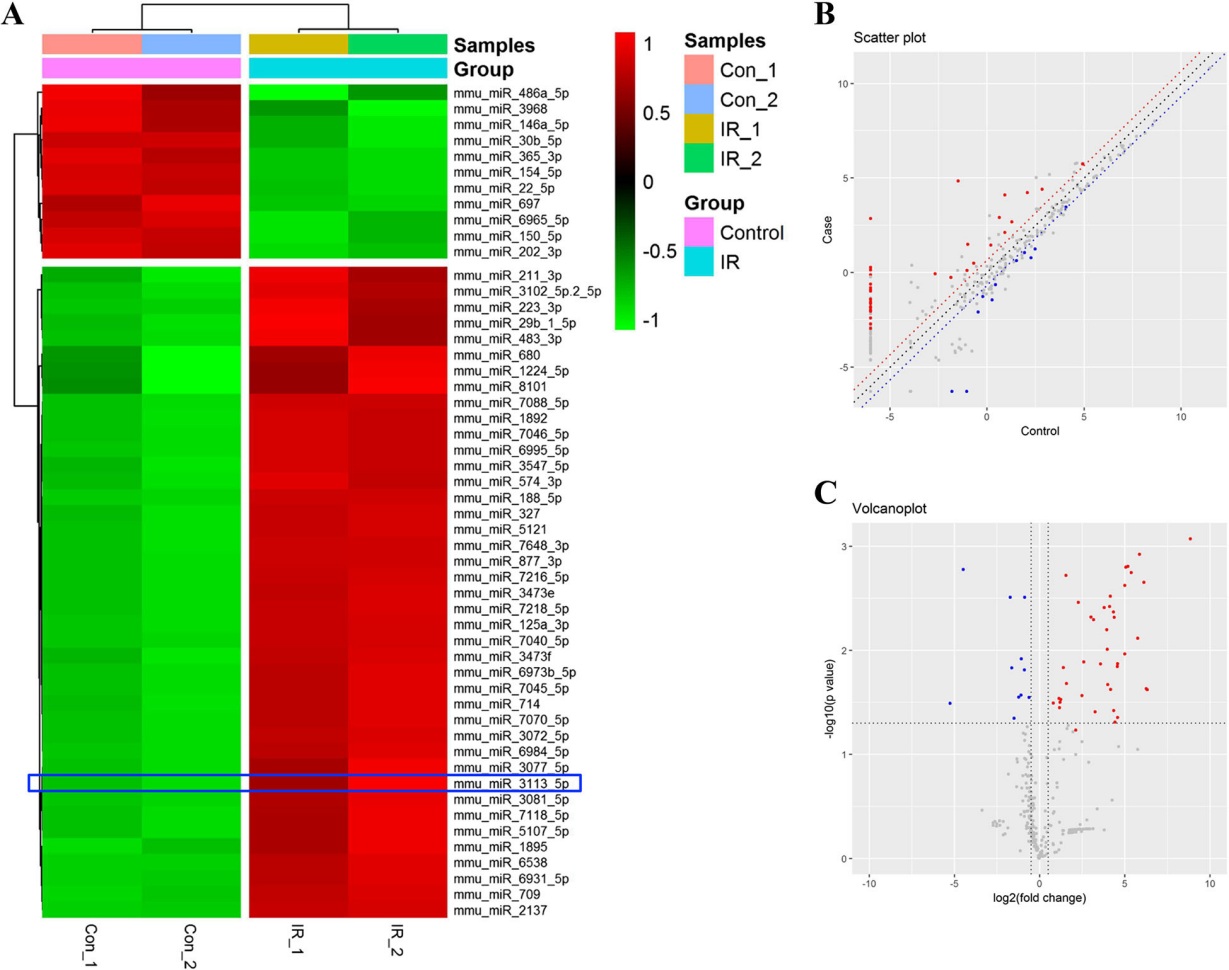
microRNA array analysis of heart tissues at 3 h after I/R injury. **a** Heatmap of the microRNAs that showed significant alteration between control (sham group) and I/R injury mice. **b-c** Scatter plot and volcano plot for all the identified microRNAs

To identify sensitive cardiac markers indicating I/R injury, we then performed a microRNA array analysis of hearts tissues with 3 h I/R injury. A total of 1882 microRNAs were identified, of which 11 were significantly down-regulated and 41 were significantly up-regulated at 3 h post reperfusion (Fig. 2a). Scatter plot analysis and volcano plot showed the distribution of all the 1882 microRNAs (Fig. 2b-c). The fold change of repression ranged from 1.6 to nearly 50, while that of upregulation varied from 1.8 to 463 (Fig. 2c). Of great interest, many of the identified microRNAs, such as microRNA 3113-5p, have been only recently discovered and remained largely unknown in function.

### Bioinformatics analysis of the differentially expressed microRNAs

**Fig. 3:**
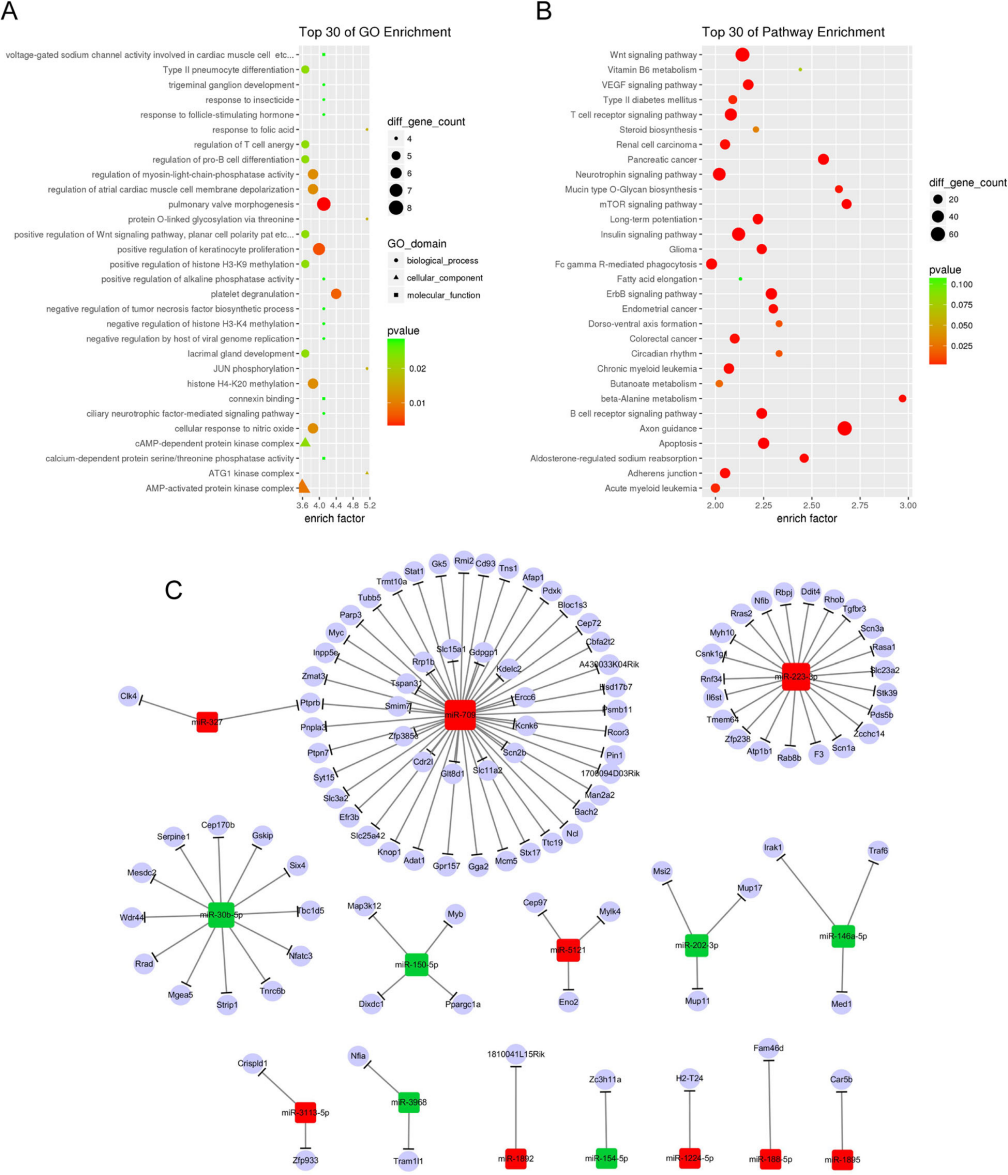
Bioinformatics analysis of the differentially expressed microRNAs. **a**-**b** Gene Ontology (GO) and Kyoto Encyclopedia of Genes and Genomes (KEGG) pathway analysis of target genes. **c** Schematic illustration of potential microRNAs target genes

Of all these differentially expressed microRNAs, target genes were predicted using four databases including TargetScan, miRDB, miRTarbase and Tarbase. Thereafter, Gene Ontology (GO) analysis revealed that biological processes included multiple processes such as regulation of myosin light chain phosphatase activity, histone H4-K20 methylation and cellular response to nitric oxide. Molecular function included connexin binding, calcium-dependent protein serine/threonine phosphatase activity and voltage-gated sodium channel activity involved in cardiac muscle cells etc. (Fig. 3a). Kyoto Encyclopedia of Genes and Genomes (KEGG) pathway analysis showed that the majority of target genes were involved in mechanistic target of rapamycin (mTOR) signaling pathway, Axon guidance, and Wnt signaling pathway etc. (Fig. 3b). Since microRNAs function mainly through targeting mRNAs [22, 23], it was hypothesized that microRNAs without valid target genes might not have biological importance. We thus refined microRNAs with target genes predicted by at least 3 databases and concentrated on microRNA 3113-5p, microRNA 5121, microRNA 327, microRNA 1892, microRNA 709, microRNA 1895, microRNA 188-5p, microRNA 1224-5p, microRNA 3968, microRNA 30b-5p, microRNA 202-3p, microRNA 154-5p, microRNA 150-5p, and microRNA 146a-5p (Fig. 3c). Target genes from 3 of the 4 databases were schematically illustrated for all the microRNAs of interest (Fig. 3c).

### RT-qPCR validation of interest microRNAs in mice

**Fig. 4:**
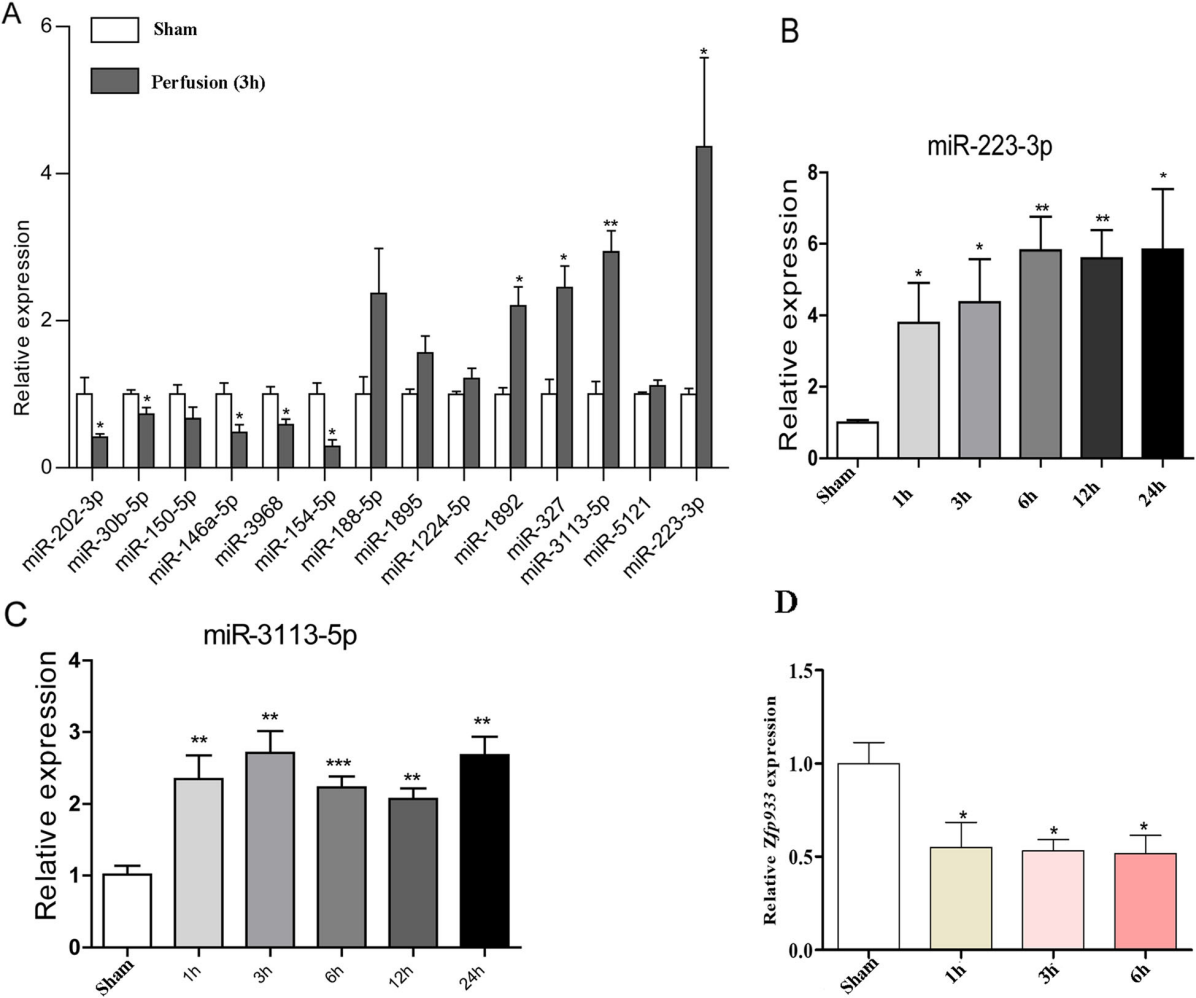
RT-qPCR validation of the differentially expressed microRNAs. **a** RT-qPCR analysis of all the 14 potential microRNAs in heart tissues. **b-c** RT-qPCR analysis of microRNA 223-3p and microRNA 3113-5p in heart tissues with distinct perfusion time. **d** RT-qPCR analysis of cardiac *Zfp933* expression (a predicted microRNA 3113-5p target gene) in heart tissues with distinct perfusion time. *, p < 0.05; **, *p* < 0.01 vs. Sham

Based on the above analysis, RT-qPCR analysis of cardiac microRNA expression was then performed. It was initially shown that microRNA 223-3p in the I/R group of mice showed the highest change by up to 4 folds as compared with Sham group of mice, while microRNA 3113-5p was only second to microRNA 223-3p that showed as high as 3-fold upregulation (Fig. 4a). Hence, we further analyzed these two microRNAs in heart tissues with distinct perfusion time. It was found that microRNA 223-3p gradually increased right after perfusion and peaked by 6 h after perfusion. It then remained relatively stable till 24 h after perfusion (Fig. 4b). Moreover, RT-qPCR validation assay showed that microRNA 3113-5p showed stable upregulation during the whole perfusion periods as compared with Sham mice hearts (Fig. 4c). Analysis of the predicted target gene of microRNA 3113-5p, zinc finger protein 933 (*Zfp933)*, showed that it was consistently repressed by 2-folds in the perfused heart tissues, independent of perfusion time (Fig. 4d).

### Circulating microRNA 3113-5p stably increased in early perfusion hours in mice

**Fig. 5:**
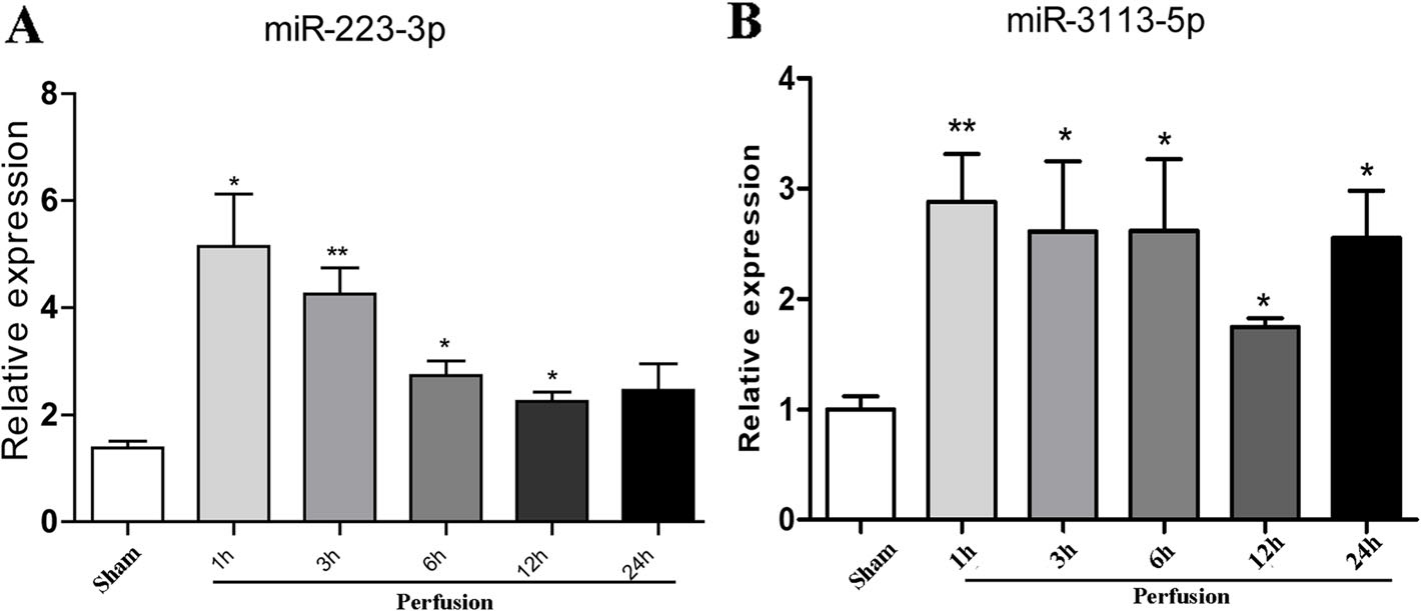
Circulating microRNA 3113-5p stably increased in the perfusion hours. **a** RT-qPCR analysis of circulating microRNA 223-3p in serum samples. **b** RT-qPCR analysis of microRNA 3113-5p in serum samples. *, p < 0.05; **, p < 0.01 vs. Sham

In addition to the cardiac expression, we also detected the circulating levels of interest microRNAs. RT-qPCR detection showed that microRNA 223-3p had maximal upregulation in serum samples of perfused mice but declined dramatically after 3 h (Fig. 5a). Unlike microRNA 223-3p, serum levels of microRNA 3113-5p remained approximately 3-fold increased throughout the whole early perfusion time (Fig. 5b).

### Circulating microRNA 3113-5p significantly increased in early I/R stimuli in human samples

**Fig. 6:**
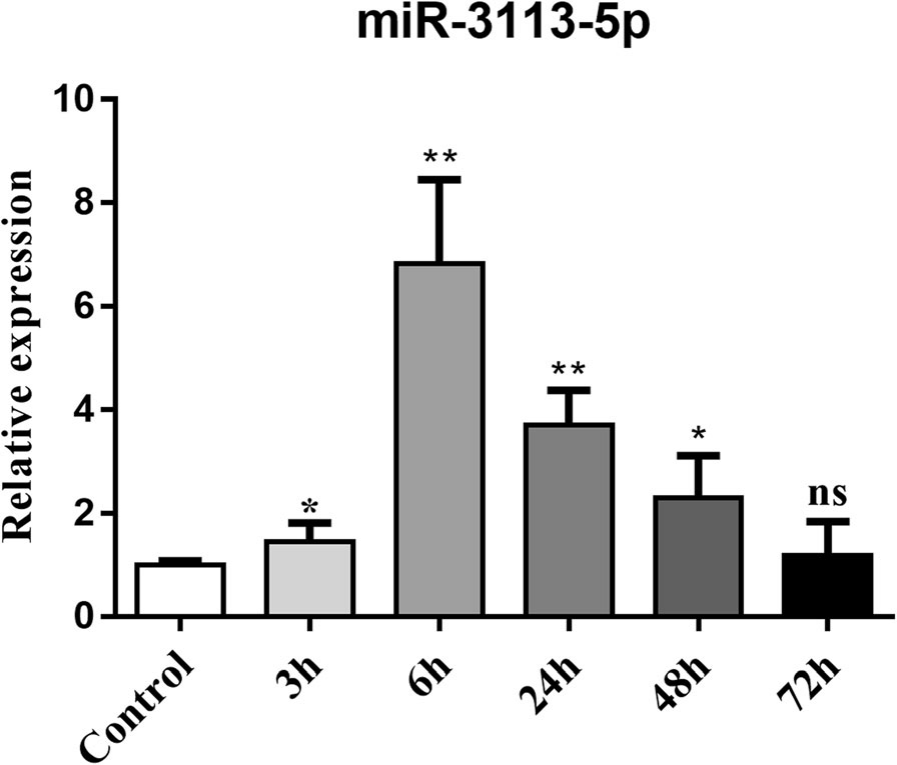
Circulating microRNA 3113-5p significantly increased in the early perfusion hours in human serum samples. Serum samples from healthy people who underwent routine health checkup served as control (Ctrl). Three patients with myocardial infraction and underwent stent implantation were collected sera during time periods of 3 h,6 h, 24 h, 48 h and 72 h. *, *P* < 0.05, ***, *P* < 0.0001 vs. Ctrl

To further detect the expression of microRNA 3113-5p in human samples, we collected serum samples from a healthy control and 3 patients with cardiac I/R injury (Table 3). All these patients were diagnosed with myocardial injury (MI) and underwent stent implantation. One hour before operation, all patients showed dramatically high levels of cardiac Troponin I (cTnI) and Myoglobin (MYO) and 1 h after operation, they still presented with higher cTnI levels than the normal upper limit (Table 3). The cardiac I/R injury patients were collected sera during time periods of 3 h, 6 h, 24 h, 48 h and 72 h. Our results showed that as soon as 3 h after cardiac I/R stimuli, the circulating microRNA 3113-5p significantly increased by up to 1.5-fold (Fig. 6). The circulating microRNA 3113-5p peaked at 6 h (6-fold) and remained significantly higher than control levels until 48 h after cardiac I/R stimuli. At 72 h after reperfusion, the serum level of microRNA 3113-5p dropped to the basal level as observed in the health control. During the early I/R injury hours (within 48 h), circulating microRNA 3113-5p levels were significantly higher than the healthy control (Fig. 6).

**Table 3:** Characteristics of the healthy control and 3 patients with MI

Case NO.	Age(y)	Gender	Pathologic diagnosis	Therapeutic measures	Myocardial enzymes			
					1 h before operation		1 h after operation	
					cTnI (ng/ml)	MYO (ng/ml)	cTnI (ng/ml)	MYO (ng/ml)
1	30	Male	NA	NA	NA	NA	NA	NA
2	82	Male	MI	Stent implantation	32.566	50.8	15.436	37.5
3	74	Male	MI	Stent implantation	9.361	485.8	18.457	20.28
4	36	Male	MI	Stent implantation	0.137	373.2	35.198	50.4

*cTnI* cardiac Troponin I (Normal reference values 0 to 0.03 ng/ml), *MYO* myoglobin (Normal reference values 17.4 to 105.7 ng/ml), *MI* myocardial infarction, *NA* not applicable

## Discussion

Organ damage insulted by I/R injury represents a serious medical condition, which often leads to deterioration or even loss of organ function [4]. I/R injury is occurred when transient tissue oxygen deprivation due to vessel occlusion accompanies with a subsequent reperfusion period following recovery of blood supply. During the reperfusion periods, multiple reactive oxygen and nitrogen species accumulate and result in eventual tissue injury [3].

In order to facilitate early diagnosis of acute I/R injury insults and risk stratification for future adverse cardiac events, it is constantly urgent to find cardiac or circulatory biomarkers that refine the diagnosis and management of patients with symptoms related to acute or chronic cardiovascular diseases [1]. microRNAs are a class of noncoding RNAs that negatively affect gene transcription through binding to the mRNA [24]. Multiple microRNAs have been identified to have diagnostic values or therapeutic potentials, but few have been applied into clinical trials. Since noncoding RNAs have been revolving to forge new ones [16], it is necessary to re-perform a microRNA array in order to discover a panel of novel microRNAs.

Since histopathological analysis revealed non-specific and non-significant changes, identification of molecular markers would be of great importance. Using an updated database with refreshed microRNAs, the current study identified 52 microRNAs that differentially expressed after 3 h of I/R injury. GO and KEGG bioinformatics analyses revealed that these microRNAs were associated with regulation of myosin light chain phosphatase activity, histone H4-K20 methylation and cellular response to nitric oxide. Many of these microRNAs were consistent with previous reports such as microRNA 146a [25] and some were inconsistent which might be explained by different I/R injured hours (we used 3 h of I/R injury for microRNA array). Of all these microRNAs, microRNA 223-3p and microRNA 3113-5p showed the highest fold changes. MicroRNA 223 expression was greatly upregulated in the livers after 75 min ischemia followed by 120 min reperfusion and its expression associated with hepatic I/R injury [26]. In a more recent study, microRNA 223-3p and -5p cooperatively suppressed necroptosis in I/R injured hearts [27]. MicroRNA 223-3p also regulated cardiac fibrosis after myocardial infarction by targeting RASA1 [9], and regulated expression of voltage-gated K+ channel Kv4.2 in acute myocardial infarction [10]. Similar with previous reports, the microRNA array also identified that microRNA-223-3p was among the top ones that altered expression, which was in conformation with previous reports that associated microRNA 223-3p with heart diseases [9, 10]. In the validation assay, cardiac microRNA 223-3p kept increasing after reperfusion but its expression in serum samples only peaked at 1 h of perfusion, indicating that microRNA 223-3p might be a valuable biomarker of cardiac I/R injury but this value was only within a very narrow time window.

Though not altered as much as microRNA 223-3p, microRNA 3113-5p was only second to it as evidenced by RT-qPCR validation. Different from microRNA 223-3p, microRNA 3113-5p was stably upregulated in both cardiac tissues and the serum samples. Right after perfusion, the cardiac microRNA 3113-5p level was approximately 2–3 folds increased and kept at this high level throughout the whole perfusion periods. Its predicted target gene, *Zfp933*, was also stably repressed and kept at a minimum level in distinct perfusion hours. Zfp933 is one member from the zinc finger protein family with its function mysterious. The C2H2-zinc finger proteins constitute the largest class of transcription factors within the human genome. These proteins are generally involved in crucial cell functions, such as survival and growth [28]. It has been reported that Zfp580, a Zfp933 homogenous protein, shows the highest expression in the heart and serves as a cardioprotection factor against cardiac I/R injury [29]. Hence, the identification of Zfp933 as the target gene of microRNA 3113-5p further implicated the functional significance of microRNA 3113-5p and its expression pattern in turn reinforced the stable cardiac increase of microRNA 3113-5p. One great advantage that makes microRNAs superior to other biomarkers is that microRNAs could stably exist in the extracellular space, such as circulating blood, urine, vitreous humor and other body fluids in spite of the existence of RNases [30]. Multiple extracellular microRNAs have been identified to have potential function and pivotal roles as disease biomarkers [31]. We therefore detected serum levels of microRNA 3113-5p in mice and in human samples in order to find non-invasive biomarkers. It was found that the serum levels of microRNA 3113-5p also stably increased ever since perfusion. The increase of microRNA 3113-5p was approximately 2–3 folds in mice serum, a change similar to that in cardiac levels, indicating that the cardiac and circulating levels of microRNA-3113-5p were highly consistent. In human serum samples, microRNA 3113-5p was significantly increased after 3 h of I/R injury and peaked by 6 h and this significant increase could be detected till 48 h. Validation of microRNA 3113-5p expression in human sera further suggested the diagnostic potential in cardiac I/R injury. Therefore, the early increase of microRNA 3113-5p made it a superior biomarker since it might serve as an early and stable indicator for cardiac I/R injury.

MicroRNA 3113-5p is a new microRNA that remains mysterious as to its function. This study represents the first one to report its functional association with cardiac I/R injury. The stable increase in cardiac and circulating microRNA 3113-5p levels suggested that it is a valuable microRNA that may aid in diagnosing I/R injury or serve as a potential therapeutic target in the heart for medical intervention of I/R injury.

## Conclusions

The present study re-analyzed the differentially expressed microRNAs during cardiac I/R injury, with an updated microRNA array. Multiple novel microRNAs that remains functionally unknown were identified. Among them, microRNA 223-3p and microRNA 3113-5p were the top ones that were validated to have functional association with I/R injury. microRNA 3113-5p is a stable biomarker that altered significantly in early hours of reperfusion. This is the first study that identified a novel microRNA, microRNA 3113-5p as a critical biomarker for cardiac I/R injury. Cardiac microRNA 3113-5p might be a useful target for therapeutic purposes and circulating microRNA 3113-5p might serve as a stable marker for diagnosis of cardiac I/R injury.

### Limitations

The present study only assessed the association of microRNA 3113-5p with cardiac I/R injury. The expression of microRNA 3113-5p in I/R injury from other organs was not detected.

## Data Availability

All data generated or analyzed during this study are included in this published article.

## Abbreviations

cTnI: cardiac Troponin I
ECG: Electrocardiogram
Gapdh: Glyceraldehyde phosphate dehydrogenase
GO: Gene ontology
HE: Hematoxylin & eosin
I/R injury: Ischemia/reperfusion injury
KEGG: Kyoto encyclopedia of genes and genomes
LAD: Descending coronary artery
MAPK: Mitogen-activated protein kinase
MI: Myocardial infarction
mTOR: mechanistic target of rapamycin
MYO: Myoglobin
NA: Not applicable
RASA1: RAS p21 protein activator 1
RT-qPCR: Reverse transcription quantitative polymerase chain reaction
STEMI: ST-elevated myocardial infarction
Zfp933: Zinc finger protein 933
